# 1,8-Bis(tos­yloxy)-9,10-anthraquinone

**DOI:** 10.1107/S1600536809051009

**Published:** 2009-12-04

**Authors:** Paweł Niedziałkowski, Damian Trzybiński, Artur Sikorski, Tadeusz Ossowski

**Affiliations:** a Faculty of Chemistry, University of Gdańsk, J. Sobieskiego 18, 80-952 Gdańsk, Poland

## Abstract

In the crystal structure of the title compound, C_28_H_20_O_8_S_2_, adjacent anthracene skeletons are parallel or inclined at an angle of 20.6 (1)°. In the mol­ecular structure, the mean plane of the anthracene skeleton makes dihedral angles of 49.6 (1) and 76.8 (1)° with the tosyl rings, and the two terminal benzene rings are oriented at an angle of 74.5 (1)° with respect to each other. The crystal structure is stabilized by inter­molecular C—H⋯O and C—O⋯π inter­actions.

## Related literature

For general background to anthraquinones, see: Cheng & Zee-Cheng (1983 ▶); Dzierzbicka *et al.* (2006 ▶); Gatto *et al.* (1996 ▶); Hunger (2003 ▶); Krapcho *et al.* (1991 ▶); Nakanishi *et al.* (2005 ▶); Zielske (1987 ▶); Zon *et al.* (2003 ▶). For related structures, see: Sereda & Akhvlediani (2003 ▶); Slouf (2002 ▶); Zain & Ng (2005 ▶). For mol­ecular inter­actions, see: Bianchi *et al.* (2004 ▶); Santos-Contreras *et al.* (2007 ▶); Spek (2009 ▶); Steiner (1999 ▶). For the synthesis, see: Ossowski *et al.* (2000 ▶).
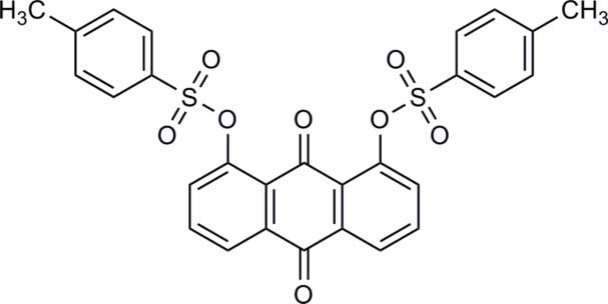


## Experimental

### 

#### Crystal data

C_28_H_20_O_8_S_2_
*M* *_r_* = 548.58
Monoclinic, 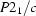

*a* = 8.263 (2) Å
*b* = 27.473 (5) Å
*c* = 11.162 (2) Å
β = 100.36 (3)°
*V* = 2492.6 (9) Å^3^
*Z* = 4
Mo *K*α radiation
μ = 0.27 mm^−1^
*T* = 295 K
0.4 × 0.3 × 0.15 mm

#### Data collection

Oxford Diffraction Gemini R ULTRA Ruby CCD diffractometer
18048 measured reflections
4371 independent reflections
3374 reflections with *I* > 2σ(*I*)
*R*_int_ = 0.050

#### Refinement

*R*[*F* ^2^ > 2σ(*F* ^2^)] = 0.058
*wR*(*F* ^2^) = 0.163
*S* = 1.15
4371 reflections
345 parameters
H-atom parameters constrained
Δρ_max_ = 0.46 e Å^−3^
Δρ_min_ = −0.32 e Å^−3^

### 

Data collection: *CrysAlis CCD* (Oxford Diffraction, 2008 ▶); cell refinement: *CrysAlis RED* (Oxford Diffraction, 2008 ▶); data reduction: *CrysAlis RED*; program(s) used to solve structure: *SHELXS97* (Sheldrick, 2008 ▶); program(s) used to refine structure: *SHELXL97* (Sheldrick, 2008 ▶); molecular graphics: *ORTEP-3* (Farrugia, 1997 ▶); software used to prepare material for publication: *SHELXL97* and *PLATON* (Spek, 2009 ▶).

## Supplementary Material

Crystal structure: contains datablocks global, I. DOI: 10.1107/S1600536809051009/xu2694sup1.cif

Structure factors: contains datablocks I. DOI: 10.1107/S1600536809051009/xu2694Isup2.hkl

Additional supplementary materials:  crystallographic information; 3D view; checkCIF report

## Figures and Tables

**Table 1:** Hydrogen-bond geometry (Å, °)

*D*—H⋯*A*	*D*—H	H⋯*A*	*D*⋯*A*	*D*—H⋯*A*
C24—H24⋯O18^i^	0.93	2.54	3.332 (4)	143
C33—H33⋯O30^ii^	0.93	2.56	3.241 (4)	130
C36—H36⋯O31^iii^	0.93	2.58	3.458 (4)	156

Symmetry codes: (i) 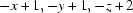; (ii) 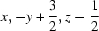; (iii) 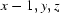.

**Table 2:** C–O⋯π inter­actions (Å,°).

C	O	*J*	O⋯*J*	C⋯*J*	C–O⋯*J*
C10	O27	*Cg*1^iv^	3.688 (3)	3.481 (3)	70.71 (17)
C10	O27	*Cg*2^v^	3.452 (3)	3.528 (3)	83.41 (18)

Symmetry codes: (iv) −*x*, −*y* + 1, −*z* + 1; (v) −*x* + 1, −*y* + 1, −*z* + 1. *Cg*1 and *Cg*2 are the centroids of the C1—C4/C11/C12 and C5—C8/C13/C14 rings respectively.

**Fig. 1.**
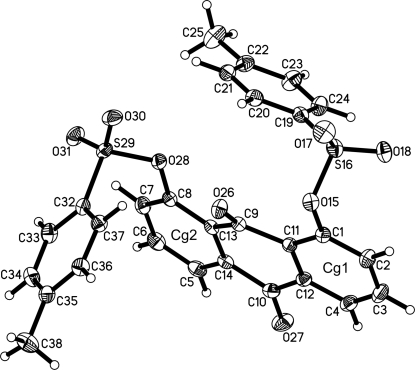
The molecular structure of the title compound showing the atom-labeling scheme. Displacement ellipsoids are drawn at the 25% probability level, and H atoms are shown as small spheres of arbitrary radius. Cg1 and Cg2 are the centroids of the C1—C4/C11/C12 and C5—C8/C13/C14 rings respectively.

**Fig. 2.**
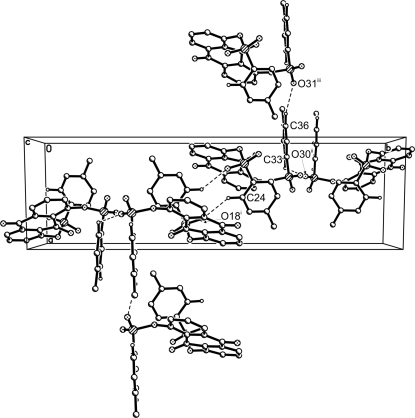
The arrangement of the molecules in the crystal structure viewed approximately along c axis. The C–H···O interactions are represented by dashed lines. H atoms not involved in interactions have been omitted. [Symmetry codes: (i) -x + 1, -y + 1, -x + 2; (ii) x, -y + 3/2, z - 1/2; (iii) x - 1, y, z.]

**Fig. 3.**
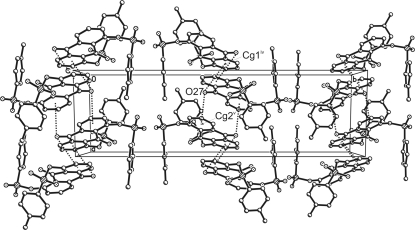
The arrangement of the molecules in the crystal structure viewed approximately along c axis. The C–O···π interactions are represented by dotted lines. H atoms not involved in interactions have been omitted. [Symmetry codes: (iv) -x, -y + 1, -z + 1; (v) -x + 1, -y + 1, -z + 1.]

## supplementary crystallographic information

### Comment

Anthraquinones, its amino and hydroxy derivatives as the largest group of naturally occurring quinines are of great practical significance in pharmacology, biochemistry and dye chemistry (Hunger, 2003). Anthraquinones are widely widespread in nature, they are occur in bark, or roots of different plants, and display various pharmacological activities such as anti-oxidant, anti-microbial, anti-fungal and anti-viral (Nakanishi *et al.*, 2005). The anthraquinone ring system is often found in antitumor drugs, such as anthracyclines, mitoxantrone, ametantrone and anthrapyrazoles (Cheng & Zee-Cheng, 1983). Its planarity allows an intercalation between base pairs of DNA in the β conformation, while its redox properties are linked to the production of radical species in biological systems. The chemical and biological activity of anthraquinone compounds depends on the different substituents of the planar ring system (Krapcho *et al.*, 1991, Gatto *et al.*, 1996). Anthraquinones are also interesting compounds for the investigations in analytical and electroanalytical chemistry due to the fact that they contain several π electrons, the reducible *p*-quinone system and are electroactive (Zon *et al.*, 2003). The tosyl group is a very good leaving group, commonly used in organic synthesis in nucleophilic substitution reaction. This phenomenon is applicable to prepare the various aminoanthraquinone from (tosy1oxy)anthraquinone precursors (Zielske, 1987). The 1,8-Bis(tosyloxy)-9,10-anthraquinone is a very convenient and often used precursor to obtain the 1,8-diaminoanthraquinones derivatives (Dzierzbicka *et al.*, 2006).

In the molecule of the title compound (Fig. 1) the bond lengths and angles characterizing the geometry of the anthraquinone skeleton are typical for this group of compounds (Sereda & Akhvlediani, 2003; Slouf, 2002; Zain & Ng, 2005).

In the packing of molecules of the title compound, the anthracene skeletons, with an average deviations from planarity of 0.044 (1) Å, are parallel or inclined at an angle of 20.1 (1)°. The mean plane of the anthracene skeleton makes dihedral angles of 49.6 (1)° and 76.8 (1)°, with the tosyl phenyl rings. Those phenyl rings are oriented at the angle of 74.5 (1)° to each other.

The crystal structure is stabilized by C–H···O (Table 1, Fig. 2) and C–O···π (Table 1, Fig. 3) intermolecular interactions. The C–H···O interactions are the hydrogen bond type (Steiner, 1999, Bianchi *et al.*, 2004). All interactions demonstrated were found by *PLATON* (Spek, 2009).

### Experimental

1,8-Bis(tosyloxy)-9,10-anthraquinone was synthesized according to the method reported in the literature (Ossowski *et al.*, 2000). To the stirring mixture of 5.0 g (20.8 mmol) 1,8-dihydroxy-9,10-anthraquinone and 5.22 g (27.4 mmol) of *p*-toluenesulfonyl chloride in 200 ml dichloromethane was dropwise added over 5 h 15 ml triethylamine in 100 ml dichloromethane. The progress of the reaction was monitored by TLC (SiO_2_, dichloromethane-petroleum ether 1:1 *v*/*v*) until the completion of reaction. The reaction mixture was stirred 6 h at room temperature. The solution was washed with water (3 *x* 100 ml), the organic phase was dried over MgSO_4_ and concentrated. The residue was purified by column chromatography on silica gel (dichloromethane-petroleum ether, 1:0.8 *v*/*v*) to afford the title compound as a yellow solid. (3.64 g, 28%). Single crystals suitable for X-ray diffraction were prepared by slow evaporation of a solution of the title compound in methanol at room temperature (m.p. = 448–450 K; elemental analysis (% found/calculated: C 61.41/61.30, H 3.65/3.67, S 11.69/11.69)).

### Refinement

H atoms were positioned geometrically, with C—H = 0.93 Å and 0.96 Å for the aromatic and methyl H atoms, respectively, and constrained to ride on their parent atoms with *U*_iso_(H) = *xU*_eq_(C), where *x* = 1.2 for the aromatic and *x* = 1.5 for the methyl H atoms.

### Crystal data

**Table:**

C_28_H_20_O_8_S_2_	*F*(000) = 1136
*M**_r_* = 548.58	*D*_x_ = 1.462 Mg m^−^^3^
Monoclinic, *P*2_1_/*c*	Mo *K*α radiation, λ = 0.71073 Å
Hall symbol: -P 2ybc	Cell parameters from 10108 reflections
*a* = 8.263 (2) Å	θ = 3.2–29.2°
*b* = 27.473 (5) Å	µ = 0.27 mm^−^^1^
*c* = 11.162 (2) Å	*T* = 295 K
β = 100.36 (3)°	Block, yellow
*V* = 2492.6 (9) Å^3^	0.4 × 0.3 × 0.15 mm
*Z* = 4	

### Data collection

**Table:**

Oxford Diffraction Gemini R ULTRA Ruby CCD diffractometer	3374 reflections with *I* > 2σ(*I*)
Radiation source: Enhance (Mo) X-ray Source	*R*_int_ = 0.050
graphite	θ_max_ = 25.1°, θ_min_ = 3.2°
Detector resolution: 10.4002 pixels mm^-1^	*h* = −9→9
ω scans	*k* = −28→32
18048 measured reflections	*l* = −13→11
4371 independent reflections	

### Refinement

**Table:**

Refinement on *F*^2^	Primary atom site location: structure-invariant direct methods
Least-squares matrix: full	Secondary atom site location: difference Fourier map
*R*[*F*^2^ > 2σ(*F*^2^)] = 0.058	Hydrogen site location: inferred from neighbouring sites
*wR*(*F*^2^) = 0.163	H-atom parameters constrained
*S* = 1.15	*w* = 1/[σ^2^(*F*_o_^2^) + (0.0997*P*)^2^ + 0.3332*P*] where *P* = (*F*_o_^2^ + 2*F*_c_^2^)/3
4371 reflections	(Δ/σ)_max_ = 0.002
345 parameters	Δρ_max_ = 0.46 e Å^−^^3^
0 restraints	Δρ_min_ = −0.32 e Å^−^^3^

### Special details

**Table:**

Geometry. All e.s.d.'s (except the e.s.d. in the dihedral angle between two l.s. planes) are estimated using the full covariance matrix. The cell e.s.d.'s are taken into account individually in the estimation of e.s.d.'s in distances, angles and torsion angles; correlations between e.s.d.'s in cell parameters are only used when they are defined by crystal symmetry. An approximate (isotropic) treatment of cell e.s.d.'s is used for estimating e.s.d.'s involving l.s. planes.

### Fractional atomic coordinates and isotropic or equivalent isotropic displacement parameters (Å^2^)

**Table:**

	*x*	*y*	*z*	*U*_iso_*/*U*_eq_	
C1	0.1135 (3)	0.52337 (10)	0.7763 (2)	0.0427 (6)	
C2	0.0657 (4)	0.47974 (11)	0.8207 (3)	0.0575 (8)	
H2	0.0301	0.4789	0.8951	0.069*	
C3	0.0707 (4)	0.43742 (11)	0.7550 (3)	0.0636 (9)	
H3	0.0365	0.4082	0.7843	0.076*	
C4	0.1262 (4)	0.43846 (11)	0.6463 (3)	0.0548 (8)	
H4	0.1311	0.4098	0.6027	0.066*	
C5	0.3761 (3)	0.52607 (12)	0.3386 (2)	0.0511 (7)	
H5	0.3765	0.4972	0.2949	0.061*	
C6	0.4458 (4)	0.56703 (12)	0.3008 (3)	0.0560 (8)	
H6	0.4921	0.5660	0.2308	0.067*	
C7	0.4480 (4)	0.60985 (11)	0.3654 (3)	0.0518 (7)	
H7	0.5004	0.6372	0.3415	0.062*	
C8	0.3724 (3)	0.61198 (10)	0.4653 (2)	0.0412 (6)	
C9	0.2008 (3)	0.57330 (9)	0.6073 (2)	0.0393 (6)	
C10	0.2362 (3)	0.48179 (10)	0.4841 (3)	0.0469 (7)	
C11	0.1676 (3)	0.52607 (9)	0.6648 (2)	0.0391 (6)	
C12	0.1754 (3)	0.48225 (9)	0.6010 (3)	0.0430 (6)	
C13	0.2964 (3)	0.57106 (9)	0.5061 (2)	0.0385 (6)	
C14	0.3048 (3)	0.52732 (10)	0.4419 (2)	0.0419 (6)	
O15	0.0972 (2)	0.56551 (7)	0.84323 (17)	0.0486 (5)	
S16	0.22980 (10)	0.57749 (3)	0.96370 (6)	0.0532 (3)	
O17	0.1499 (3)	0.61401 (9)	1.0209 (2)	0.0762 (7)	
O18	0.2768 (3)	0.53273 (8)	1.02503 (19)	0.0653 (6)	
C19	0.3976 (3)	0.60173 (10)	0.9090 (2)	0.0449 (6)	
C20	0.3917 (4)	0.64943 (11)	0.8680 (3)	0.0541 (8)	
H20	0.3000	0.6687	0.8712	0.065*	
C21	0.5222 (4)	0.66780 (11)	0.8228 (3)	0.0540 (8)	
H21	0.5183	0.6998	0.7955	0.065*	
C22	0.6606 (4)	0.63985 (11)	0.8167 (3)	0.0546 (7)	
C23	0.6631 (4)	0.59232 (11)	0.8589 (3)	0.0644 (9)	
H23	0.7547	0.5730	0.8561	0.077*	
C24	0.5337 (4)	0.57324 (11)	0.9046 (3)	0.0582 (8)	
H24	0.5376	0.5413	0.9325	0.070*	
C25	0.8010 (5)	0.66019 (14)	0.7636 (4)	0.0856 (12)	
H25A	0.8561	0.6342	0.7297	0.128*	
H25B	0.8771	0.6762	0.8264	0.128*	
H25C	0.7595	0.6832	0.7007	0.128*	
O26	0.1427 (3)	0.61106 (7)	0.63596 (19)	0.0530 (5)	
O27	0.2365 (3)	0.44406 (8)	0.4261 (2)	0.0714 (7)	
O28	0.3778 (2)	0.65378 (6)	0.53713 (16)	0.0471 (5)	
S29	0.34049 (9)	0.70699 (2)	0.48060 (7)	0.0481 (2)	
O30	0.3284 (3)	0.73551 (8)	0.5843 (2)	0.0638 (6)	
O31	0.4611 (3)	0.71850 (8)	0.4092 (2)	0.0643 (6)	
C32	0.1493 (3)	0.70184 (10)	0.3851 (3)	0.0444 (6)	
C33	0.1394 (4)	0.70045 (11)	0.2604 (3)	0.0556 (8)	
H33	0.2343	0.7015	0.2266	0.067*	
C34	−0.0135 (4)	0.69754 (12)	0.1866 (3)	0.0586 (8)	
H34	−0.0206	0.6962	0.1026	0.070*	
C35	−0.1561 (4)	0.69661 (10)	0.2349 (3)	0.0525 (7)	
C36	−0.1428 (4)	0.69827 (11)	0.3605 (3)	0.0533 (7)	
H36	−0.2380	0.6979	0.3941	0.064*	
C37	0.0083 (3)	0.70044 (10)	0.4368 (3)	0.0488 (7)	
H37	0.0156	0.7010	0.5209	0.059*	
C38	−0.3217 (5)	0.69311 (16)	0.1525 (4)	0.0821 (11)	
H38A	−0.3199	0.7114	0.0795	0.123*	
H38B	−0.4047	0.7061	0.1936	0.123*	
H38C	−0.3458	0.6596	0.1320	0.123*	

### Atomic displacement parameters (Å^2^)

**Table:**

	*U*^11^	*U*^22^	*U*^33^	*U*^12^	*U*^13^	*U*^23^
C1	0.0381 (14)	0.0445 (15)	0.0461 (15)	0.0029 (11)	0.0092 (11)	0.0010 (12)
C2	0.0600 (18)	0.0551 (18)	0.0611 (19)	−0.0041 (14)	0.0213 (15)	0.0089 (15)
C3	0.065 (2)	0.0446 (17)	0.083 (2)	−0.0099 (14)	0.0186 (18)	0.0101 (16)
C4	0.0511 (16)	0.0393 (15)	0.072 (2)	−0.0036 (12)	0.0054 (15)	−0.0027 (14)
C5	0.0485 (16)	0.0628 (19)	0.0408 (16)	0.0076 (13)	0.0051 (12)	−0.0107 (13)
C6	0.0539 (17)	0.077 (2)	0.0393 (16)	0.0067 (15)	0.0131 (13)	−0.0016 (14)
C7	0.0536 (16)	0.0576 (18)	0.0457 (16)	0.0011 (13)	0.0127 (13)	0.0055 (13)
C8	0.0388 (13)	0.0450 (15)	0.0383 (14)	0.0039 (11)	0.0029 (11)	0.0020 (11)
C9	0.0376 (13)	0.0422 (14)	0.0372 (14)	0.0016 (11)	0.0045 (11)	−0.0010 (11)
C10	0.0436 (14)	0.0440 (16)	0.0504 (16)	0.0015 (11)	0.0017 (12)	−0.0101 (13)
C11	0.0345 (13)	0.0406 (14)	0.0412 (14)	0.0019 (10)	0.0046 (10)	0.0015 (11)
C12	0.0365 (13)	0.0406 (15)	0.0504 (16)	0.0005 (11)	0.0041 (11)	−0.0014 (12)
C13	0.0364 (13)	0.0456 (14)	0.0321 (13)	0.0053 (11)	0.0029 (10)	0.0001 (11)
C14	0.0381 (13)	0.0480 (15)	0.0375 (14)	0.0056 (11)	0.0010 (11)	−0.0053 (12)
O15	0.0510 (11)	0.0515 (11)	0.0462 (11)	0.0089 (8)	0.0163 (9)	0.0006 (8)
S16	0.0651 (5)	0.0582 (5)	0.0396 (4)	0.0068 (3)	0.0181 (3)	−0.0027 (3)
O17	0.0945 (18)	0.0811 (17)	0.0624 (14)	0.0122 (13)	0.0391 (13)	−0.0169 (12)
O18	0.0802 (15)	0.0693 (14)	0.0469 (12)	0.0063 (11)	0.0126 (11)	0.0156 (10)
C19	0.0543 (16)	0.0428 (15)	0.0372 (14)	0.0068 (12)	0.0070 (12)	−0.0058 (11)
C20	0.0512 (17)	0.0468 (16)	0.0612 (19)	0.0107 (13)	0.0018 (14)	−0.0073 (14)
C21	0.0632 (19)	0.0402 (15)	0.0557 (18)	0.0016 (13)	0.0025 (15)	−0.0025 (13)
C22	0.0587 (18)	0.0450 (16)	0.0602 (18)	−0.0005 (13)	0.0111 (15)	−0.0095 (13)
C23	0.060 (2)	0.0445 (18)	0.092 (3)	0.0124 (14)	0.0231 (17)	−0.0039 (16)
C24	0.066 (2)	0.0404 (16)	0.070 (2)	0.0106 (14)	0.0173 (16)	0.0004 (14)
C25	0.084 (3)	0.064 (2)	0.118 (3)	−0.0084 (19)	0.044 (2)	−0.004 (2)
O26	0.0669 (13)	0.0389 (11)	0.0587 (12)	0.0081 (9)	0.0259 (10)	0.0001 (9)
O27	0.0901 (17)	0.0553 (13)	0.0714 (15)	−0.0082 (11)	0.0213 (13)	−0.0257 (12)
O28	0.0543 (11)	0.0439 (11)	0.0429 (11)	−0.0036 (8)	0.0085 (8)	0.0006 (8)
S29	0.0439 (4)	0.0417 (4)	0.0596 (5)	−0.0070 (3)	0.0114 (3)	0.0020 (3)
O30	0.0678 (14)	0.0496 (12)	0.0720 (14)	−0.0069 (10)	0.0070 (11)	−0.0169 (10)
O31	0.0490 (12)	0.0625 (14)	0.0855 (16)	−0.0099 (10)	0.0230 (11)	0.0141 (12)
C32	0.0452 (15)	0.0402 (14)	0.0500 (16)	0.0007 (11)	0.0143 (12)	0.0055 (12)
C33	0.0544 (17)	0.0613 (18)	0.0564 (18)	0.0063 (14)	0.0241 (14)	0.0098 (14)
C34	0.069 (2)	0.0629 (19)	0.0436 (16)	0.0113 (15)	0.0102 (15)	0.0056 (14)
C35	0.0535 (17)	0.0416 (15)	0.0605 (19)	0.0073 (12)	0.0050 (14)	0.0021 (13)
C36	0.0437 (16)	0.0584 (18)	0.0600 (19)	0.0032 (13)	0.0156 (14)	0.0024 (14)
C37	0.0483 (15)	0.0535 (17)	0.0469 (16)	−0.0007 (12)	0.0144 (12)	0.0011 (13)
C38	0.066 (2)	0.099 (3)	0.073 (2)	0.010 (2)	−0.0094 (18)	−0.001 (2)

### Geometric parameters (Å, °)

**Table:**

C1—C2	1.382 (4)	C19—C20	1.386 (4)
C1—O15	1.398 (3)	C20—C21	1.367 (5)
C1—C11	1.398 (4)	C20—H20	0.9300
C2—C3	1.379 (5)	C21—C22	1.389 (4)
C2—H2	0.9300	C21—H21	0.9300
C3—C4	1.372 (5)	C22—C23	1.387 (4)
C3—H3	0.9300	C22—C25	1.502 (5)
C4—C12	1.394 (4)	C23—C24	1.369 (5)
C4—H4	0.9300	C23—H23	0.9300
C5—C6	1.364 (5)	C24—H24	0.9300
C5—C14	1.387 (4)	C25—H25A	0.9600
C5—H5	0.9300	C25—H25B	0.9600
C6—C7	1.379 (4)	C25—H25C	0.9600
C6—H6	0.9300	O28—S29	1.6000 (19)
C7—C8	1.373 (4)	S29—O30	1.416 (2)
C7—H7	0.9300	S29—O31	1.419 (2)
C8—O28	1.397 (3)	S29—C32	1.745 (3)
C8—C13	1.403 (4)	C32—C33	1.380 (4)
C9—O26	1.210 (3)	C32—C37	1.390 (4)
C9—C13	1.491 (4)	C33—C34	1.381 (4)
C9—C11	1.495 (4)	C33—H33	0.9300
C10—O27	1.223 (3)	C34—C35	1.381 (5)
C10—C12	1.479 (4)	C34—H34	0.9300
C10—C14	1.485 (4)	C35—C36	1.387 (4)
C11—C12	1.406 (4)	C35—C38	1.508 (4)
C13—C14	1.407 (4)	C36—C37	1.380 (4)
O15—S16	1.608 (2)	C36—H36	0.9300
S16—O17	1.415 (2)	C37—H37	0.9300
S16—O18	1.427 (2)	C38—H38A	0.9600
S16—C19	1.745 (3)	C38—H38B	0.9600
C19—C24	1.378 (4)	C38—H38C	0.9600
			
C2—C1—O15	117.8 (3)	C21—C20—C19	119.2 (3)
C2—C1—C11	121.6 (3)	C21—C20—H20	120.4
O15—C1—C11	120.6 (2)	C19—C20—H20	120.4
C3—C2—C1	120.2 (3)	C20—C21—C22	121.7 (3)
C3—C2—H2	119.9	C20—C21—H21	119.2
C1—C2—H2	119.9	C22—C21—H21	119.2
C4—C3—C2	119.9 (3)	C23—C22—C21	117.7 (3)
C4—C3—H3	120.0	C23—C22—C25	121.3 (3)
C2—C3—H3	120.0	C21—C22—C25	121.0 (3)
C3—C4—C12	120.3 (3)	C24—C23—C22	121.5 (3)
C3—C4—H4	119.8	C24—C23—H23	119.3
C12—C4—H4	119.8	C22—C23—H23	119.3
C6—C5—C14	120.2 (3)	C23—C24—C19	119.5 (3)
C6—C5—H5	119.9	C23—C24—H24	120.2
C14—C5—H5	119.9	C19—C24—H24	120.2
C5—C6—C7	120.6 (3)	C22—C25—H25A	109.5
C5—C6—H6	119.7	C22—C25—H25B	109.5
C7—C6—H6	119.7	H25A—C25—H25B	109.5
C8—C7—C6	119.7 (3)	C22—C25—H25C	109.5
C8—C7—H7	120.1	H25A—C25—H25C	109.5
C6—C7—H7	120.1	H25B—C25—H25C	109.5
C7—C8—O28	121.9 (2)	C8—O28—S29	122.72 (16)
C7—C8—C13	121.7 (3)	O30—S29—O31	119.70 (14)
O28—C8—C13	116.2 (2)	O30—S29—O28	102.71 (12)
O26—C9—C13	121.7 (2)	O31—S29—O28	108.68 (12)
O26—C9—C11	121.2 (2)	O30—S29—C32	110.80 (14)
C13—C9—C11	116.9 (2)	O31—S29—C32	108.96 (14)
O27—C10—C12	120.5 (3)	O28—S29—C32	104.82 (11)
O27—C10—C14	120.6 (3)	C33—C32—C37	121.0 (3)
C12—C10—C14	118.8 (2)	C33—C32—S29	120.1 (2)
C1—C11—C12	117.2 (2)	C37—C32—S29	118.9 (2)
C1—C11—C9	122.8 (2)	C32—C33—C34	119.0 (3)
C12—C11—C9	119.8 (2)	C32—C33—H33	120.5
C4—C12—C11	120.8 (3)	C34—C33—H33	120.5
C4—C12—C10	118.7 (3)	C33—C34—C35	121.4 (3)
C11—C12—C10	120.5 (2)	C33—C34—H34	119.3
C8—C13—C14	116.9 (2)	C35—C34—H34	119.3
C8—C13—C9	122.8 (2)	C34—C35—C36	118.4 (3)
C14—C13—C9	120.2 (2)	C34—C35—C38	120.5 (3)
C5—C14—C13	120.8 (3)	C36—C35—C38	121.1 (3)
C5—C14—C10	119.1 (2)	C37—C36—C35	121.5 (3)
C13—C14—C10	120.1 (2)	C37—C36—H36	119.2
C1—O15—S16	120.01 (16)	C35—C36—H36	119.2
O17—S16—O18	120.23 (15)	C36—C37—C32	118.6 (3)
O17—S16—O15	102.67 (14)	C36—C37—H37	120.7
O18—S16—O15	108.09 (12)	C32—C37—H37	120.7
O17—S16—C19	110.62 (15)	C35—C38—H38A	109.5
O18—S16—C19	109.39 (14)	C35—C38—H38B	109.5
O15—S16—C19	104.49 (12)	H38A—C38—H38B	109.5
C24—C19—C20	120.4 (3)	C35—C38—H38C	109.5
C24—C19—S16	120.0 (2)	H38A—C38—H38C	109.5
C20—C19—S16	119.6 (2)	H38B—C38—H38C	109.5
			
O15—C1—C2—C3	176.5 (3)	C12—C10—C14—C13	−6.3 (4)
C11—C1—C2—C3	0.1 (4)	C2—C1—O15—S16	77.9 (3)
C1—C2—C3—C4	1.3 (5)	C11—C1—O15—S16	−105.6 (2)
C2—C3—C4—C12	−0.9 (5)	C1—O15—S16—O17	−165.1 (2)
C14—C5—C6—C7	0.8 (4)	C1—O15—S16—O18	−37.1 (2)
C5—C6—C7—C8	−3.2 (4)	C1—O15—S16—C19	79.4 (2)
C6—C7—C8—O28	176.9 (2)	O17—S16—C19—C24	148.7 (2)
C6—C7—C8—C13	1.9 (4)	O18—S16—C19—C24	14.1 (3)
C2—C1—C11—C12	−1.6 (4)	O15—S16—C19—C24	−101.4 (2)
O15—C1—C11—C12	−177.9 (2)	O17—S16—C19—C20	−32.5 (3)
C2—C1—C11—C9	172.5 (2)	O18—S16—C19—C20	−167.1 (2)
O15—C1—C11—C9	−3.9 (4)	O15—S16—C19—C20	77.3 (2)
O26—C9—C11—C1	−19.9 (4)	C24—C19—C20—C21	0.2 (4)
C13—C9—C11—C1	165.1 (2)	S16—C19—C20—C21	−178.6 (2)
O26—C9—C11—C12	154.0 (3)	C19—C20—C21—C22	0.2 (5)
C13—C9—C11—C12	−21.0 (3)	C20—C21—C22—C23	−0.4 (5)
C3—C4—C12—C11	−0.7 (4)	C20—C21—C22—C25	178.3 (3)
C3—C4—C12—C10	179.4 (3)	C21—C22—C23—C24	0.3 (5)
C1—C11—C12—C4	1.9 (4)	C25—C22—C23—C24	−178.4 (4)
C9—C11—C12—C4	−172.4 (2)	C22—C23—C24—C19	0.0 (5)
C1—C11—C12—C10	−178.1 (2)	C20—C19—C24—C23	−0.3 (5)
C9—C11—C12—C10	7.6 (4)	S16—C19—C24—C23	178.5 (3)
O27—C10—C12—C4	3.1 (4)	C7—C8—O28—S29	47.3 (3)
C14—C10—C12—C4	−173.8 (2)	C13—C8—O28—S29	−137.4 (2)
O27—C10—C12—C11	−176.9 (2)	C8—O28—S29—O30	169.05 (19)
C14—C10—C12—C11	6.3 (4)	C8—O28—S29—O31	−63.2 (2)
C7—C8—C13—C14	1.6 (4)	C8—O28—S29—C32	53.2 (2)
O28—C8—C13—C14	−173.7 (2)	O30—S29—C32—C33	144.9 (2)
C7—C8—C13—C9	−174.4 (2)	O31—S29—C32—C33	11.2 (3)
O28—C8—C13—C9	10.3 (3)	O28—S29—C32—C33	−105.0 (2)
O26—C9—C13—C8	22.0 (4)	O30—S29—C32—C37	−33.5 (3)
C11—C9—C13—C8	−163.1 (2)	O31—S29—C32—C37	−167.2 (2)
O26—C9—C13—C14	−154.0 (2)	O28—S29—C32—C37	76.6 (2)
C11—C9—C13—C14	21.0 (3)	C37—C32—C33—C34	−0.1 (4)
C6—C5—C14—C13	2.8 (4)	S29—C32—C33—C34	−178.5 (2)
C6—C5—C14—C10	−177.4 (3)	C32—C33—C34—C35	0.8 (5)
C8—C13—C14—C5	−4.0 (3)	C33—C34—C35—C36	−0.5 (4)
C9—C13—C14—C5	172.2 (2)	C33—C34—C35—C38	−179.6 (3)
C8—C13—C14—C10	176.3 (2)	C34—C35—C36—C37	−0.5 (4)
C9—C13—C14—C10	−7.6 (3)	C38—C35—C36—C37	178.6 (3)
O27—C10—C14—C5	−2.9 (4)	C35—C36—C37—C32	1.1 (4)
C12—C10—C14—C5	173.9 (2)	C33—C32—C37—C36	−0.8 (4)
O27—C10—C14—C13	176.9 (2)	S29—C32—C37—C36	177.5 (2)

### Hydrogen-bond geometry (Å, °)

**Table:**

*D*—H···*A*	*D*—H	H···*A*	*D*···*A*	*D*—H···*A*
C24—H24···O18^i^	0.93	2.54	3.332 (4)	143
C33—H33···O30^ii^	0.93	2.56	3.241 (4)	130
C36—H36···O31^iii^	0.93	2.58	3.458 (4)	156

Symmetry codes: (i) −*x*+1, −*y*+1, −*z*+2; (ii) *x*, −*y*+3/2, *z*−1/2; (iii) *x*−1, *y*, *z*.

### Table 2 C–O···π interactions (Å,°).

**Table:**

C	O	*J*	O···*J*	C···*J*	C–O···*J*
C10	O27	Cg1^iv^	3.688 (3)	3.481 (3)	70.71 (17)
C10	O27	Cg2^v^	3.452 (3)	3.528 (3)	83.41 (18)

Symmetry codes: (iv) -x, -y+1, -z+1; (v) -x+1, -y+1, -z+1. *Cg*1 and *Cg*2 are the centroids of the C1-C4/C11/C12 and C5-C8/C13/C14 rings respectively.
